# Obstructive Sleep Apnea and Postoperative Cognitive Decline in Non‐Cardiac Surgery: A Prospective Cohort Study

**DOI:** 10.1002/brb3.71154

**Published:** 2025-12-23

**Authors:** Sakura Kinjo, Junyong In, Eunjung Lim

**Affiliations:** ^1^ Department of Anesthesia and Perioperative Care University of California San Francisco California U.S.A; ^2^ Department of Anesthesiology and Pain Medicine Dongguk University Ilsan Hospital Goyang Republic of Korea; ^3^ Department of Quantitative Health Sciences, John A Burns School of Medicine University of Hawaii Honolulu Hawaiʻi U.S.A

**Keywords:** depression, obstructive sleep apnea, postoperative cognitive dysfunction, sleep monitoring

## Abstract

**Background:**

Obstructive sleep apnea may be linked to postoperative cognitive decline. We conducted a prospective observational study to determine whether the severity of sleep apnea is associated with postoperative cognitive decline following non‐cardiac surgery.

**Methods:**

Adult patients undergoing elective non‐cardiac surgery were enrolled in the study. A series of cognitive tests, including the verbal fluency test, digit symbol substitution test, and word list recall, were used to compare cognitive function before and after surgery. The Telephone Interview for Cognitive Status assessed long‐term cognitive function at 30 and 90 days post‐surgery.

**Results:**

In total, 65 patients were included in the analysis, with a mean age of 63.7 years (SD = 7.4). The overall incidence of postoperative cognitive decline within the first 2 days after surgery was 18.5%. Our study found that patients with moderate to severe obstructive sleep apnea did not have a higher risk of postoperative cognitive decline compared to those with no or mild obstructive sleep apnea (*p* = 0.339). However, a history of depression emerged as an independent risk factor for postoperative cognitive decline (*p* = 0.045). Furthermore, there was no significant difference in cognitive function between the obstructive sleep apnea groups at 30 and 90 days after surgery.

**Conclusions:**

Our study indicates no clear association between the severity of obstructive sleep apnea and postoperative cognitive decline within 90 days after surgery. Instead, we identified preoperative depression as a significant risk factor for postoperative cognitive decline. Therefore, it is important to exercise caution when managing patients with depression.

## Graphical abstract

This study found no clear link between the severity of obstructive sleep apnea and postoperative cognitive decline within 90 days after surgery. However, preoperative depression was identified as a significant risk factor for postoperative cognitive decline.

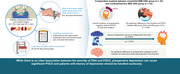

## 1 Introduction

Obstructive sleep apnea (OSA) is a sleep‐breathing disorder characterized by repetitive partial or complete airway obstruction during sleep. OSA is highly prevalent in the United States. A recent study in 2024 (Sonmez et al. 2025) estimated that 83.7 million adults have OSA (overall 32.4% prevalence), with 59% being male and 41% being female in the United States. In this study, the distribution of OSA severity was estimated to be 52% mild, 30% moderate, and 18% severe. OSA is associated with multiple co‐morbidities such as hypertension, coronary artery disease, atrial fibrillation, stroke, and diabetes (Yeghiazarians et al. 2021). Additionally, patients with OSA are at risk of developing neuropsychological deficits, such as memory deficits, cognitive decline, and mood disorders (Bubu et al. 2020; Gupta and Simpson 2015). Animal studies (Feng et al. 2012; Miyo et al. 2025; Sapin et al. 2015) indicate that chronic continuous or intermittent hypoxia causes hippocampal impairment, a region important for learning and memory. Some neuroimaging studies (Canessa et al. 2011; Macey et al. 2008) have also shown that hypoxia leads to structural changes in the brains of individuals suffering from OSA.

Patients with OSA face a high risk of postoperative oxygen desaturation in the perioperative period, particularly when opioids are administered (Cozowicz et al. 2018; Kaw et al. 2012). A study has shown that postoperative oxygen desaturation may occur at rates 12–14 times higher in OSA patients receiving oral or intravenous opioids compared to those treated with OSA with non‐opioid analgesic agents (Bolden et al. 2007). Patients are at the highest risk, typically during the acute and sub‐acute postoperative periods, especially during the rebound phase of rapid eye movement (REM) sleep (Rosenberg‐Adamsen et al. 1996). Changes in sleep architecture associated with these hypoxic episodes may contribute to postoperative neurocognitive disorder (PND) such as postoperative delirium (POD) and postoperative cognitive decline/dysfunction (POCD) (Wang et al. 2021).

POCD is a common occurrence after major surgery, particularly in older patients. POCD is associated with increased mortality (Monk et al. 2008), can persist for a substantial period after surgery, and results in a significantly decreased quality of life (Steinmetz et al. 2009). POCD often can be subtle and may only be detected by changes in neurocognitive testing conducted before and after surgery. Various neurocognitive tests (e.g., Mini‐Mental State Examination (MMSE), Montreal Cognitive Assessment, Cambridge Neuropsychological Test Automated Battery, Digit Symbol Substitution Test (DSST)) have been used to diagnose POCD; however, no gold standard test currently exists. The underlying etiology/pathophysiology requires further clarification. While numerous studies have examined the prevalence and risk factors associated with POCD (Canet et al. 2003; Sun et al. 2024), the impact of OSA on long‐term cognitive outcomes after surgery remains unclear. This study aimed to investigate whether OSA influences cognitive function after surgery and, if so, which cognitive domains are affected. We hypothesize that patients with OSA have an increased risk of POCD.

## 2 Methods

The prospective observational cohort study was conducted at a single university hospital from March 2014 to June 2018. The Institutional Review Board at the University of California, San Francisco approved the study (No.12‐08773). Written informed consent was obtained from each study patient before surgery.

### 2.1 Patients

This study enrolled patients undergoing elective non‐cardiac surgery who were expected to stay in the hospital postoperatively for more than 48 h. The inclusion criteria were that the patient be 50 years or older and fluent in English. Patients with a history of dementia, tremors, peripheral vascular disease, severe cardiovascular disease, and severe visual and auditory handicaps were excluded. Additionally, surgeries involving the brain, airway, or lungs were not included in the study.

The preoperative interview was conducted by a trained research assistant in the preoperative anesthesia clinic, typically less than 2 weeks before surgery. General demographic data, including age, sex, race, level of education and the patient's health information (American Society of Anesthesiologists physical classification, use of continuous positive airway pressure, preoperative comorbid conditions (hypertension, stroke, transitional ischemic attack, depression, diabetes, coronary artery disease, pulmonary disease) and medications, which may affect sleep and cognition (opioids, benzodiazepine, antidepressants, antihistamine) were obtained preoperatively. In addition, potential covariates (pain levels and depression) were also gathered. Increased postoperative pain disrupts sleep, while poor sleep intensifies pain sensitivity (Xu et al. 2024). Poor pain control may increase the risk of POCD (Khaled et al. 2025). Therefore, pain levels were assessed both before and after surgery using the 11‐point numeric rating scale, with the average of current, minimum, and maximum pain levels recorded. Depression is known to be a strong factor linked to OSA and sleep (Jackson et al. 2011). Furthermore, our previous study (Kinjo et al. 2012) and others (Lee et al. 2024) demonstrated that preoperative depression was associated with increased postoperative pain. In addition, a recent meta‐analysis (Diep et al. 2024) suggests that preoperative depression is associated with a greater risk of POD. We used the Center for Epidemiologic Studies Depression Scale (CES‐D) (Radloff 1977) to assess patients' mood. The CES‐D is a widely used depression scale that has been studied in the OSA population (Bardwell et al. 2000). The CES‐D consists of 20 questions, including various aspects of depression, such as mood, sleep, appetite, and feeling. The total scores range from 0 to 60. The scores of 0 to 15 are considered to have no evidence of depression, 16 to 29 is mild depression, 30–44 is moderate depression, and 45–60 is severe depression. A series of neurocognitive tests was conducted, and other covariates, including intraoperative factors (the type of surgery and its duration), were obtained through a review of medical charts (Varpaei et al. 2024).

### 2.2 Assessment of OSA and Measurement of Sleep

Patients were oriented to a sleep monitoring device (WatchPAT, Itamar Medical, Caesarea, Israel). This device was used for two nights before surgery in the patients' homes and two nights after surgery. Key variables obtained from the device included the preoperative Apnea–Hypopnea index (pAHI), which was used to assess the severity of OSA. OSA was categorized as follows: none/mild OSA was defined as a pAHI of fewer than 15 events per hour; moderate OSA was characterized by a pAHI of at least 15 events per hour but fewer than 30 events per hour; severe OSA was defined as 30 events per hour or more. The patients were subsequently divided into two groups: none or mild OSA (Group NM) and moderate or severe OSA (Group MS).

### 2.3 Cognitive Function Assessment

Cognitive function was assessed using a battery of neuropsychological tests in the preoperative evaluation clinic. This study utilized three neurocognitive tests to evaluate various aspects of cognitive function during the acute postoperative period. The tests included a verbal fluency test, a digit symbol substitution test (DSST), and a word list test. Additionally, the Telephone Interview for Cognitive Status (TICS) was used preoperatively, as well as 30 and 90 days postoperatively.

The verbal fluency test measures a patient's ability to organize words into meaningful clusters. The participant is given a letter of the alphabet and has 60 s to name as many words as possible that start with that letter, excluding proper nouns and the exact words with different tenses. This test indirectly assesses short‐term memory, as the individual must keep track of the words that have already been mentioned.

The DSST evaluates attention and psychomotor speed, with scores ranging from 0 to 90. Patients are presented with nine symbols that correspond to single‐digit numbers. Following this, they receive a list of single‐digit numbers and must write the corresponding symbol in the appropriate box under each number in the order they appear.

For the word list test, patients are given a list of nine related words and are asked to recall as many of them as possible over three trials, with 5‐min intervals between each trial. This test measures short‐term retention and learning capabilities.

The TICS measures global cognitive ability by assessing key domains such as orientation, attention, language, and memory (including learning and recall). It is an 11‐item test, with a maximum score of 41 points, that correlates highly with the MMSE and is widely used for dementia and mild cognitive impairment screening (Brandt 1988).

During the patient's hospital stay, a member of the research team followed up with each patient to assess their sleep and pain levels and administered a series of neurocognitive tests in the patient's room for the first two postoperative days (PDs). If the patient was discharged on PD2, the patient was contacted via phone to assess their sleep quality, pain levels, and mental functions. In this situation, the DSST was excluded since it requires an in‐person assessment.

A significant cognitive decline was indicated by changes of ≥7 in the verbal fluency test, ≥7 in the DSST, and ≥4 in the word list (Sands et al. 2000; Wang et al. 2007). Based on a previous study (Wang et al. 2007), POCD was defined as a patient showing a notable decline from their baseline performance on two or more neurocognitive tests on either PD1 or PD2. If only two of the three tests were performed and there was a decline in both, it was considered POCD.

### 2.4 Sample Size Calculation

The exact incidence of POCD in OSA patients is not well established. We estimated the sample size based on published results on the association between sleep apnea and cognitive dysfunction by Wagner et al. (2018), who assessed the relationship between sleep apnea and cognitive dysfunction. Since the study did not report standard deviations for cognitive changes in the cognitive tests (DemTect test and Digit span forward test), we calculated effect sizes (≥ 0.85) for the difference between the control group and the OSA group. Two‐sample *t*‐tests indicated that a minimum of 28 patients per group (a total of 56) is needed to detect such an effect size with a significance level of 0.05 and a power of 0.9.

To account for potential incomplete data, we added more than 10 % of patients to the calculated sample size.

### 2.5 Statistical Analysis

Descriptive statistics were calculated to summarize the preoperative and intraoperative variables, as well as the results of the neurocognitive tests. To assess their bivariate associations with preoperative OSA, chi‐squared tests or Fisher's exact tests were used for categorical variables, while two‐sample *t*‐tests or Wilcoxon non‐parametric tests were used for continuous variables. A multivariable logistic regression was conducted, including bivariate variables with a *p*‐value less than 0.10. The results were presented using odds ratios (OR) and 95% confidence intervals (CI). Model fit was assessed using the area under the receiver operating curve (AUC) and the Hosmer–Lemeshow test. Variance inflation factors (VIF) were calculated to assess multicollinearity, with VIFs greater than 5 indicating serious multicollinearity. As a sensitivity analysis, multiple imputation was performed using 20 imputed datasets, applying the same logistic regression model to address missing data. All analyses were conducted using R version 4.2.3 (R Foundation for Statistical Computing, Vienna, Austria), and a *p*‐value less than 0.05 was considered statistically significant. In addition, a post hoc power analysis was performed using G*Power (Faul et al. 2009) to estimate the sample size required to detect a significant effect of preoperative OSA on cognitive decline, based on the OR identified in the multivariable logistic regression model.

## 3 Results

**FIGURE 1:**
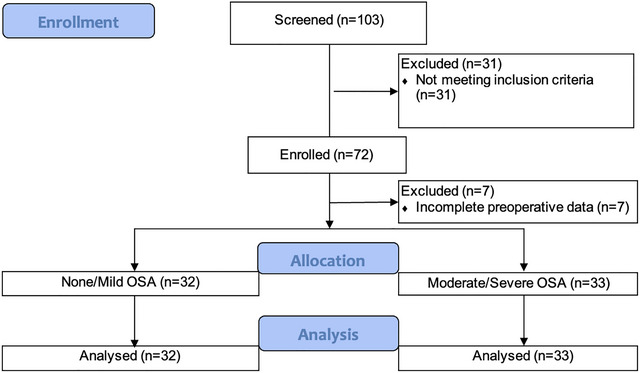
Consort flow diagram. OSA: obstructive sleep apnea.

Seven out of 72 patients were excluded from the study due to missing preoperative data, resulting in a total of 65 patients included in the analysis (see Figure 1). The mean age of the participants was 63.7 years (standard deviation = 7.4, range: 50–82), with approximately equal numbers of males and females. Many of the patients underwent orthopedic surgery (44 patients, 67.7%), followed by urologic surgery (10 patients, 15.4%), spine surgery (4 patients, 6.2%), general surgery (2 patients, 3.1%), gynecologic surgery (2 patients, 3.1%), otolaryngology (1 patient, 1.5%), ophthalmology (1 patient, 1.5%), and combined general/urology procedures (1 patient, 1.5%).

Among the patients, 15 had severe OSA, 18 had moderate OSA, and 32 had none or mild OSA (refer to Table 1). There were no significant differences in demographic data, including age, sex, race, level of education, ASA physical status, and the use of CPAP, between the two groups. Co‐morbidities and clinical variables between the two OSA groups were similar. On CES‐D, the highest score was 44, which translates to moderate depression. Additionally, intraoperative variables showed no significant differences. Table 2 presents the results of cognitive tests, comparing each cognitive assessment between the two OSA groups. The baseline cognitive functions were similar across both groups. When comparing preoperative and postoperative values within each OSA group for various tests, no significant differences were found.

**TABLE 1:** Patients’ characteristics.

Variable	Total (*n* = 65)	Preoperative OSA status		*p*
		Group NM (*n* = 32)	Group MS (*n* = 33)	
*Demographic data*				
Age (years)	63.7 ± 7.4	64.4 ± 6.7	63.1 ± 8.0	0.471
Sex, male	35 (53.8%)	18 (54.5%)	17 (53.1%)	0.909
Race, White	14 (21.5%)	6 (18.2%)	8 (25.0%)	0.504
Education, college, or higher	48 (73.8%)	23 (71.9%)	25 (75.8%)	0.722
ASA, III/IV	15 (23.1%)	9 (27.3%)	6 (18.8%)	0.415
Use of CPAP	8 (12.3%)	4 (12.1%)	4 (12.5%)	>0.999
*Underlying disease*				
Hypertension	29 (44.6%)	16 (48.5%)	13 (40.6%)	0.524
Stroke	2 (3.1%)	2 (6.1%)	0 (0.0%)	0.492
TIA	3 (4.6%)	2 (6.1%)	1 (3.1%)	>0.999
CES‐D				0.421
No (0–15)	45 (77.6%)	23 (82.1%)	22 (73.3%)	
Mild/moderate (16–44)	12 (22.4%)	5 (17.9%)	8 (26.7%)	
Self‐reported history of depression	21 (32.3%)	9 (27.3%)	12 (37.5%)	0.378
Diabetes	6 (9.2%)	3 (9.1%)	3 (9.4%)	>0.999
CAD	3 (4.6%)	2 (6.1%)	1 (3.1%)	>0.999
Pulmonary disease	11 (16.9%)	6 (18.2%)	5 (15.6%)	0.783
*Preoperative medication*				
Opioids	16 (24.6%)	8 (24.2%)	8 (25.0%)	0.943
Benzodiazepine	6 (9.2%)	3 (9.1%)	3 (9.4%)	>0.999
Antidepressants	11 (16.9%)	5 (15.2%)	6 (18.8%)	0.699
Antihistamines	3 (4.6%)	2 (6.1%)	1 (3.1%)	>0.999
*Preoperative pain (NRS)*	2.7 ± 2.3	2.7 ± 2.4	2.8 ± 2.3	0.928
*Intraoperative variable*				
Surgery type, orthopedic^a^	44 (67.7%)	25 (75.8%)	19 (59.4%)	0.158
Surgery time (min)	156.5 ± 113.4	142.6 ± 89.7	170.9 ± 133.5	0.321

Abbreviations: CES‐D, Center for Epidemiologic Studies Depression Scale; DSST, Digit Symbol Substitution Test; TICS, Telephone Interview for Cognitive Status; AHI, Apnea–Hypopnea Index; CPAP, continuous positive airway pressure; TIA, transient ischemic attack; CAD, coronary artery disease; ASA, American Society of Anesthesiologists Physical Status; OSA, obstructive sleep apnea; NM, no/mild obstructive sleep apnea group; MS, moderate/severe obstructive sleep apnea group; NRS, Numerical Rating Scale.

*Note*: Data are presented as mean ± standard deviation or number (%). *p*‐value was computed using Fisher's exact test or Chi‐square test for categorical variables and two‐sample *t*‐test for continuous variables.

**^a^** One patient had two types of surgeries—general and urology.

**TABLE 2:** Preoperative OSA status and neurocognitive tests.

Cognitive test	Day	Total (*n* = 65)	Preoperative OSA status		*p*
			Group NM (*n* = 32)	Group MS (*n* = 33)	
Verbal Fluency	Preoperative	13.2 ± 4.4	12.5 ± 4.0	13.8 ± 4.8	0.236
	PD1	12.9 ± 4.4	12.1 ± 3.8	13.8 ± 4.9	0.117
	PD2	13.1 ± 5.2	11.7 ± 4.6	14.5 ± 5.5	0.051
	∆_pre,PD1_	0.3 ± 3.5	0.6 ± 3.5	0.0 ± 3.5	0.545
	∆_pre,PD2_	0.6 ± 4.3	1.4 ± 4.9	−0.1 ± 3.6	0.230
DSST	Preoperative	49.9 ± 12.4	48.1 ± 13.7	51.6 ± 10.8	0.256
	PD1	45.9 ± 12.9	45.4 ± 12.1	46.3 ± 13.8	0.767
	PD2	47.9 ± 15.6	48.1 ± 13.5	47.7 ± 18.0	0.937
	∆_pre,PD1_	4.2 ± 10.8	3.0 ± 9.2	5.3 ± 12.2	0.400
	∆_pre,PD2_	0.6 ± 14.2	0.8 ± 11.3	0.3 ± 17.2	0.928
Word list	Preoperative	21.1 ± 3.2	20.5 ± 3.3	21.8 ± 3.1	0.125
	PD1	19.3 ± 3.3	19.0 ± 3.1	19.6 ± 3.6	0.459
	PD2	19.9 ± 3.8	18.9 ± 3.6	20.8 ± 3.8	0.064
	∆_pre,PD1_	1.9 ± 2.9	1.6 ± 3.1	2.2 ± 2.6	0.436
	∆_pre,PD2_	1.3 ± 3.7	1.9 ± 4.6	0.7 ± 2.5	0.244
TICS	Preoperative	34.9 ± 2.6	34.5 ± 2.5	35.3 ± 2.6	0.193
	PD30	35.2 ± 3.6	34.6 ± 3.6	35.7 ± 3.5	0.307
	PD90	36.5 ± 3.1	36.7 ± 2.7	36.4 ± 3.5	0.806
	∆_pre,PD30_	−0.3 ± 2.8	0.2 ± 2.9	−0.7 ± 2.7	0.274
	∆_pre,PD90_	−1.6 ± 2.8	−1.6 ± 3.2	−1.6 ± 2.4	>0.999
POCD		12 (18%)	8 (24%)	4 (12%)	0.339

Abbreviations: DSST, Digit Symbol Substitution Test; OSA, obstructive sleep apnea; PD, postoperative day; TICS, Telephone Interview for Cognitive Status; NM, no/mild obstructive sleep apnea group; MS, moderate/severe obstructive sleep apnea group; POCD, postoperative cognitive decline; ∆_pre,PD1_, the difference between preoperative value and PD1 value.

*Note*: Data are presented as mean ± standard deviation. *p*‐value was computed using a two‐sample *t*‐test.

Overall, 18% of the patients (12 out of 65) experienced POCD either on PD1 or PD2. Specifically, 10.9% of the patients (7 out of 64) exhibited POCD on PD1, while 11.3% (6 out of 53) showed POCD on PD2. There was no significant difference in the incidence of POCD between the two groups: 24% in Group NM compared to 12% in Group MS, *p* = 0.339 (Table 2).

We also examined whether any demographic, clinical, or intraoperative variables were associated with POCD. Among all the variables assessed, only self‐reported history of depression was significantly associated with POCD, with 58.3% of the 12 patients experiencing it (7 out of 12 patients), *p* = 0.045 (Table 3).

**TABLE 3:** Patients’ characteristics and postoperative cognitive decline.

Variable	Total (*n* = 65)	Postoperative cognitive decline		*p*
		No (*n* = 53)	Yes (*n* = 12)	
*Demographic*				
Age (years)	63.7 ± 7.4	63.8 ± 7.3	63.6 ± 8.0	0.941
Sex, male	35 (53.8%)	29 (54.7%)	6 (50.0%)	0.767
Race, White	14 (21.5%)	11 (20.8%)	3 (25.0%)	0.711
Education, college, or higher	48 (73.8%)	38 (71.7%)	10 (83.3%)	0.494
ASA, III/IV	15 (23.1%)	11 (20.8%)	4 (33.3%)	0.449
Use of CPAP	8 (12.3%)	5 (9.4%)	3 (25.0%)	0.158
*Underlying disease*				
Hypertension	29 (44.6%)	21 (39.6%)	8 (66.7%)	0.089
Stroke	2 (3.1%)	2 (3.8%)	0 (0.0%)	>0.999
TIA	3 (4.6%)	3 (5.7%)	0 (0.0%)	>0.999
CES‐D				0.101
No (0–15)	45 (77.6%)	39 (83.0%)	6 (54.5%)	
Mild/moderate (16–44)	13 (22.4%)	8 (17.0%)	5 (45.5%)	
Self‐reported history of depression	21 (32.1%)	14 (26.4%)	7 (58.3%)	**0.045**
Diabetes	6 (9.2%)	5 (9.4%)	1 (8.3%)	>0.999
CAD	3 (4.6%)	2 (3.8%)	1 (8.3%)	0.464
Pulmonary disease	11 (16.9%)	8 (15.1%)	3 (25.0%)	0.412
*Preoperative medication*				
Opioid	16 (24.6%)	12 (22.6%)	4 (33.3%)	0.470
Benzodiazepine	6 (9.2%)	4 (7.5%)	2 (16.7%)	0.305
Antidepressant	11 (16.9%)	9 (17.0%)	2 (16.7%)	>0.999
Antihistamine	3 (4.6%)	3 (5.7%)	0 (0.0%)	>0.999
*Preoperative pain (NRS)*	2.7 ± 2.3	2.7 ± 2.3	2.9 ± 2.5	0.830
*Intraoperative variable*				
Surgery type, orthopedic^a^	44 (67.7%)	37 (69.8%)	7 (58.3%)	0.503
Surgery time (min)	156.5 ± 113.4	148.1 ± 106.2	193.9 ± 140.0	0.304

Abbreviations: CES‐D, Center for Epidemiologic Studies Depression Scale; DSST, Digit Symbol Substitution Test; TICS, Telephone Interview for Cognitive Status; AHI, Apnea–Hypopnea Index; CPAP, continuous positive airway pressure; TIA, transient ischemic attack; CAD, coronary artery disease; ASA, American Society of Anesthesiologists Physical Status; OSA, obstructive sleep apnea; NM, None/mild obstructive sleep apnea group; MS, moderate/severe obstructive sleep apnea group; NRS: Numerical Rating Scale.

*Note*: Data are presented as mean ± standard deviation or number (%). *p*‐value was computed using Fisher's exact test or chi‐square test for categorical variable and two sample *t*‐test for continuous variable.

**^a^** One patient had two types of surgeries—general and urology.

Table 4 presents the results of the multivariable logistic regression. No evidence of multicollinearity was found (VIFs < 1.5), and the model demonstrated satisfactory fit, with an AUC of 0.756 (95% CI = 0.609–0.923) and a non‐significant Hosmer–Lemeshow test (*p* = 0.950). Consistent with the bivariate analyses, only self‐reported history of depression was significantly associated with POCD, with an OR of 4.24 (95% CI: 1.09–18.25). Preoperative OSA was not statistically significant; however, patients who exhibited moderate or severe preoperative OSA had 2.62 times higher odds of developing POCD compared to those with no or mild OSA. A post hoc power analysis indicated that a sample size of 216 patients would be required to detect a significant effect of preoperative OSA on cognitive decline under conditions similar to those of the current study. Finally, the results from multiple imputation were consistent with those from the complete‐case analysis (see Table 4).

**TABLE 4:** Multivariable logistic regression on postoperative cognitive decline.

	Complete‐case analysis			Multiple imputation
Variable	OR (95% CI)	*p*	VIF	OR (95% CI)
Preoperative OSA			1.05	
None/mild	Ref			Ref
Moderate/severe	2.62 (0.66, 12.19)	0.185		2.62 (0.61, 11.25)
Self‐reported history of depression	4.24 (1.08, 18.25)	0.041	1.05	4.24 (1.03, 17.45)
Hypertension	2.57 (0.66, 11.24)	0.182	1.01	2.57 (0.62, 10.58)

Abbreviations: CI, confidence interval; OR, odds ratio; OSA, obstructive sleep apnea; Ref, reference; VIF, variance inflation factor.

*Note*: Area under the receiver operating curve = 0.766, 95% CI = (0.609, 0.923).

## 4 Discussion

We conducted a prospective cohort study investigating the relationship between OSA and POCD in elective non‐cardiac surgery. Our findings suggest that the severity of OSA does not significantly affect the risk of POCD during the acute postoperative period or in the months following surgery. While previous studies have linked OSA to cognitive dysfunction, particularly in non‐surgical contexts, our results imply that the perioperative setting may not exacerbate this risk.

Numerous studies have investigated the relationship between OSA and long‐term cognitive decline or Alzheimer's disease (AD) outside of perioperative settings. For instance, a review by Bubu et al. (2020) highlighted that OSA is associated with mild cognitive impairment or AD, particularly in older patients experiencing sleep disturbances.

### 4.1 OSA and PND (POD and POCD)

Some studies (Dooijeweerd et al. 2022; Fadayomi et al. 2018; Sun et al. 2022; Xiao et al. 2024) have demonstrated that OSA is independently associated with an increased risk of POD. Meta‐analyses indicate that patients with OSA have up to twofold higher odds of developing POD compared to those without OSA, and this finding is consistent across various surgical populations. The underlying mechanisms are thought to involve intermittent hypoxia, sleep fragmentation, and systemic inflammation, which contribute to neurocognitive vulnerability. Additionally, perioperative factors such as opioid sensitivity and postoperative oxygen desaturation may exacerbate delirium risk in this population.

Studies have shown that patients with OSA are at an increased risk for POCD, particularly after major surgeries such as cardiac surgery (Greaves et al. 2019) joint replacement surgery (Wu et al. 2022). Although it is plausible that OSA increases the risk of persistent cognitive impairment, direct evidence is still limited due to fewer studies examining the impact of OSA on POCD and the nature of studies; many are observational and retrospective analyses. Existing studies have produced conflicting results. For example, Wu et al. (2022) conducted a retrospective analysis of cognition in elderly patients who underwent joint replacement, evaluating cognition using the MMSE. They found no difference in baseline MMSE scores between patients with and without OSA before surgery; however, patients with OSA had lower MMSE scores at discharge, 1 month, and 1 year after surgery. In contrast, Wagner et al. (2018) conducted an observational pilot study on patients who underwent surgery with intravenous anesthesia and found that those at high risk of OSA exhibited less impairment in memory function and working memory performance. The authors speculated that this could be due to the beneficial effect of intrinsic hypoxic preconditioning in these patients.

More recent studies align with our findings (Devinney et al. 2025; Wu et al. 2023). Devinney et al. (2025) investigated the relationship between untreated OSA and POCD in patients aged 60 or older undergoing non‐cardiac surgery, concluding that OSA did not significantly affect the incidence or severity of POCD at 6 weeks or 1 year postoperatively. Similarly, Wu et al. (2023) found that moderate to high OSA risk (as determined by the STOP‐BANG questionnaire) was not associated with POCD in patients undergoing gastrointestinal surgery unless they also exhibited excessive daytime sleepiness.

### 4.2 Preoperative Depression and POCD

Another significant finding in our study is that preoperative depression was linked to an increased risk of POCD. The recent meta‐analysis has shown that preoperative depression is a significant risk factor for delirium (Diep et al. 2024). In their study, for patients with preoperative depression, the risk of delirium was 1.91 times greater compared with patients without preoperative depression.

However, it is still unknown whether depression influences cognition beyond the immediate postoperative period. Previous research on this topic has produced mixed results (Kadoi et al. 2011; Lewis et al. 2022). For instance, Kadoi et al. (2011) identified preoperative depression as an independent predictor for both short‐term (7 days) and long‐term (6 months) POCD in diabetic patients after cardiac surgery. Conversely, Lewis et al. (2022) found no significant association between preoperative depression and POCD 1 month after surgery. Given these inconsistencies, further research is needed to clarify the relationship between depression and POCD. However, it is worth noting that depression and POCD may share common risk factors, including age, chronic medical conditions, and systemic inflammation (Kudoh et al. 2001; Monk et al. 2008; Vink et al. 2008). A multidisciplinary approach that incorporates thorough preoperative assessment, optimization of preoperative care, and postoperative monitoring may benefit both conditions.

Our study has several limitations. First, some patients were discharged early, which prevented them from completing cognitive tests that required in‐person assessments. As a result, the incidence of POCD may have been underestimated. Secondly, we categorized the severity of OSA into two groups (none/mild vs. moderate/severe) for the purpose of comparison. To more accurately examine the relationship between different severity levels of OSA and POCD, a larger sample size is necessary to analyze patients with mild, moderate, and severe OSA separately. Thirdly, POCD can persist for several weeks to years. We followed patients for up to 90 days after their surgery; thus, we may have missed valuable longitudinal data. Fourth, while we attempted to assess multiple cognitive domains using various cognitive tests, it is possible that we overlooked some areas. Lastly, our study identified self‐reported preoperative depression as an independent risk factor for POCD. The Comprehensive Evaluation of Depressive Symptoms (CED‐S) indicated that there were no severely depressed patients in our study. It is possible that if we had included severely depressed patients, the effects on cognition could have been more pronounced. Future research should investigate the duration of depression and its severity in relation to cognitive outcomes.

Our study also has several strengths. Unlike studies that rely solely on questionnaires to assess OSA risk, we used a portable sleep monitoring device (WatchPAT) to measure sleep parameters directly. Furthermore, we employed multiple cognitive tests to evaluate different cognitive domains, rather than depending on a single general test such as the MMSE, which may lack the sensitivity needed to detect subtle cognitive changes.

## 5 Conclusions

Our study found no clear association between OSA severity and POCD in the acute postoperative setting or at 30 and 90 days postoperatively. However, preoperative depression has been identified as an independent risk factor for POCD. These findings highlight the importance of comprehensive preoperative evaluations, including mental health assessments. Future research should focus on prospective and large‐scale studies to explore these associations further and identify potential interventions to mitigate the risk of POCD.

## Author Contributions

**Sakura Kinjo**: writing – original draft, conceptualization, investigation. **Junyong In**: conceptualization, validating, writing – review and editing. **Eunjung Lim**: conceptualization, writing – review and editing, data curation and analysis.

## Funding

EL was partially supported by National Institues of Health grants U54GM138062 (PICO) and U54MD007601 (Ola HAWAII). The study also received support from University of California San Francisco internal grants, including the Mount Zion Health Fund and the Department of Anesthesia Seed Support for Clinical Research Award.

## Conflicts of Interest

The authors declare no conflicts of interest.

## Ethics Statement

The authors have nothing to report.

## Data Availability

The data that support the findings of this study are available from the corresponding author upon reasonable request.
